# Evaluation of root canal morphology in permanent maxillary and mandibular anterior teeth in Saudi subpopulation using two classification systems: a CBCT study

**DOI:** 10.1186/s12903-022-02187-1

**Published:** 2022-05-10

**Authors:** Azhar Iqbal, Mohmed Isaqali Karobari, Mohammad Khursheed Alam, Osama Khattak, Sultan Metab Alshammari, Abdul Habeeb Adil, Tahir Yusuf Noorani, Hamoud Ali Algarani, Meshal Aber Alonazi, Kumar Chandan Sirivastava, Rakhi Issrani

**Affiliations:** 1 Department of Restorative Dentistry, College of Dentistry, Jouf University, Sakaka, 72345 Saudi Arabia; 2 Conservative Dentistry Unit, School of Dental Sciences, Universiti Sains Malaysia, Health Campus, 16150 Kubang Kerian, Kota Bharu, Kelantan Malaysia; 3 Department of Restorative Dentistry and Endodontics, Faculty of Dentistry, University of Puthisastra, Phnom Penh, 12211 Cambodia; 4 Preventive Dentistry Department, College of Dentistry, Jouf University, Sakaka, 72345 Saudi Arabia; 5 Center for Transdisciplinary Research (CFTR), Saveetha Dental College, Saveetha Institute of Medical and Technical Sciences, Saveetha University, Chennai, India; 6 Department of Public Health, Faculty of Allied Health Sciences, Daffodil international University, Dhaka, Bangladesh; 7 Department of Community Dentistry, School of Dental Sciences, Universiti Sains Malaysia, Health Campus, 16150 Kubang Kerian, Kota Bharu, Kelantan Malaysia; 8 Oral Medicine and Radiology, Department of Oral and Maxillofacial Surgery and Diagnostic Sciences, College of Dentistry, Jouf University, Sakaka, 72345 Saudi Arabia

**Keywords:** Root canal, Morphology, Classification, Dental anatomy, Endodontics, Dental Pulp

## Abstract

**Background:**

Adequate knowledge of root canal morphology and possible variations is essential to achieve perfect root canal treatment and overcome treatment failure. Appropriate knowledge on root and canal morphology, communication, and documentation amongst dentists will be challenging from a diagnostic and successful treatment point of view.

**Methods:**

A total of 3420 samples were included in this study from 285 cone-beam computed tomography images of the Saudi residents, including 171 males and 114 females aged 15 to 68 years from retrospective data dated from January 2018 to April 2021. The images were examined in sagittal, axial and coronal views using a 3D version software 1.0.10.6388. The number of canal and canal morphology was recorded using Vertucci and the new classification system. The SPSS 26 was used to conduct the statistical analysis as descriptive statistics such as mean; standard deviation and frequency were calculated. The Chi-square test analysed the data with the significance level set at 0.05.

**Results:**

A total of 285 subjects participated in the study. Majority of the participants were Saudi nationals (80.7%), followed by Indian (7.4%), Pakistani (4.2%) and other nationalities. According to Vertucci and the new classification system, Type I and ^1^TN^1^ were the most common types, followed by Type III and Type IV, and then ^1^TN^1-2-1^ and ^1^TN^1-2^ in mandibular anteriors. The prevalence of canal variations in mandibular canine was higher in females than in males (*P* = 0.002). Maxillary laterals and mandibular anteriors showed the significant difference in the prevalence of root canal variation in relation to the ethnicity (*P* = 0.001) and age of the patients. Younger patients showed more variations than the older patients (*P* = 0.012, *P* = 0.023, *P* = 0.001, *P* = 0.001) in terms of maxillary laterals, mandibular central, laterals and canines, respectively.

**Conclusion:**

Mandibular permanent anteriors showed a wide range of canal variations and canal complexity. Males and females did not demonstrate a wide range of variation in the root canal morphology except for the canines in relation to the gender of the patients.

## Background

Most root canal treatment failures and inadequacies in endodontics are due to the limited acquaintance of the diverse anatomy of roots and variations in canal morphology [1]. Locating, disinfecting with appropriate canal preparation, and further sealing all the available canals will ultimately ensure the tooth’s good prognosis and health. Practitioners today are savvy about the conventional canal anatomy of teeth. Although the obturation and filling of the other canals are perfect, adequate knowledge of root and root canal morphological characteristics and possible variations is required to achieve perfect endodontic treatment and overcome treatment failure [1–3]. The permanent mandibular anterior teeth have reported extra roots or root canal variations compared with the maxillary anteriors. We can expect differences in canal morphologies in maxillary teeth with anomalies, such as palato–gingival groove and dens invaginatus [4].

Literature showed that the morphology in the maxillary anterior teeth was commonly present with single root and single canal, whereas mandibular anterior teeth revealed double canals [5, 6]. In addition, populations of diverse ethnic backgrounds play a role in the variation in canal morphology [4]. The morphological diversities in mandibular incisors (central and lateral) were revealed by the study conducted by Perlea et al. [7]; they stated that out of the mandibular incisors teeth, 81% had a single canal, and the other 19% had two canals, mostly varying from Weine’s class 1 to 4 [7]. Furthermore, literature shows higher prevalence of two canals in mandibular incisors [5, 8], which vary according to ethnicity, gender and age [9, 10]. Recently conducted study amongst Malaysian population revealed that mandibular anteriors showed a wide range of canal variations, and the canal complexity is significantly affected by sex, ethnicity and age [4]. Different populations also influence the complexity of the root canal morphology of anterior teeth and several studies conducted amongst Turkish, Chinese, Iranian, Jordanian and American populations revealed variations in root canal morphology of permanent anterior teeth [11–16].

To assess the morphology of the root and canal, various methods have been used, such as staining, sectioning, decalcification and clearing technique and conventional radiographs [17–20]. Furthermore, the radiographic technique with 3D images has been utilised, such as micro-computed tomography (MicroCT) and cone-beam computed tomography (CBCT). The method utilised in the current study for root and canal evaluation was CBCT to account for a non-invasive and meticulous study of the morphology. It does not interfere with the morphology of the tooth structure in any way and allows us to determine the minute anatomy of the canal structure. CBCT, different from 2D radiography, permits a 3D visualisation of the root and canal morphologies [4].

The classification introduced by Vertucci and the new classification system are used for classifying the root and canal morphology proposed by Ahmed et al. [21–23]. This new classification accounts for the various intercanal complexities that the original Vertucci’s classification, and its supplementation method might not be clarified in detail. According to several studies, the classification of the internal anatomy of several tooth types has discrepancies; for example, maxillary premolar teeth with three canals. Vertucci, Seelig [24] classified this variation as type VIII and defined it as three separate, distinct root canals extending from the pulp chamber to the apex. However, no information is provided in the classification to describe whether these canals are encased in single- or multi-rooted teeth. As a result, in most studies, three-canalled single-/double-/three-rooted maxillary premolars were still referred to as type VIII configuration [22, 25, 26]. Clearly, in terms of clinical management of teeth undergoing root canal treatment, defining the number of roots, rather than only canals, is critical, because it has implications for accessing cavity preparation, mechanical instrumentation and root canal filling procedures [27].

The current study used CBCT scan to examine root canal morphology of maxillary and mandibular anterior teeth of the Saudi subpopulation according to Vertucci’s classification and the new classification system for classifying the root canal morphology. The inclusion of maxillary and mandibular anterior teeth in the current study is important because both teeth have some anatomical differences in root canal morphology, resulting in difficulties in radiographic interpretation of their apical third, as well as a doubtful prognosis after the completion of root canal treatment. The current study aims to evaluate the root and the canal morphology in the permanent anterior teeth amongst Saudi sab population using CBCT. Furthermore, the variation in the root canal morphology in relation to the gender, age and the ethnicity of the patients was analysed.

## Methods

The Local Committee of Bioethics for Research provided ethical clearance for this study with the Ethical Approval No 09-04/41. Informed consent was waived by the Committee of Bioethics for Research, College of Dentistry, Jouf University, Sakaka, Saudi Arabia, due to the retrospective nature of the study. In addition, the patients sign a general consent before any treatment or investigation is rendered, including consent to use the findings in future retrospective studies without any personal identification. The sample size of 183 scans was determined using the G power 3.1.9.4 software with x^2^ test, goodness-of-fit test was statistical test, and the type of power analysis was A priori. The required sample size was computed given α, power and effect size. The present study includes 3420 permanent maxillary and mandibular anterior teeth from 285 CBCT images of the patients, including 171 males and 114 females aged 15 to 68 years from retrospective data dated from January 2018 to April 2021. The patient’s records were accessed with prior permission from the dean and the hospital director, and the patients’ age, gender and ethnicity were recorded. The CBCT images were accessed through the radiology department of dental school, and the images were obtained for purposes other than this study. The patients’ name and personal data were not recorded to ensure the protection of any identifiable data on the CBCT scans that could interfere with ethical responsibilities towards patient’s data; access to the recorded data was only for the research team.

The study required CBCT images of the mandibular and maxillary anterior teeth (central, lateral incisors and canines) to be included. Images with the mandibular and maxillary anterior healthy teeth, with only minor caries or restorations in the crown, having fully developed root apex, and images without the radiographic defects were included in the study. The current study excluded root canal-treated teeth, teeth with post and core, crowns, fractured maxillary and mandibular anteriors, teeth with resorption defects, calcification, and teeth with crown and root anomalies.

**Fig. 1:**
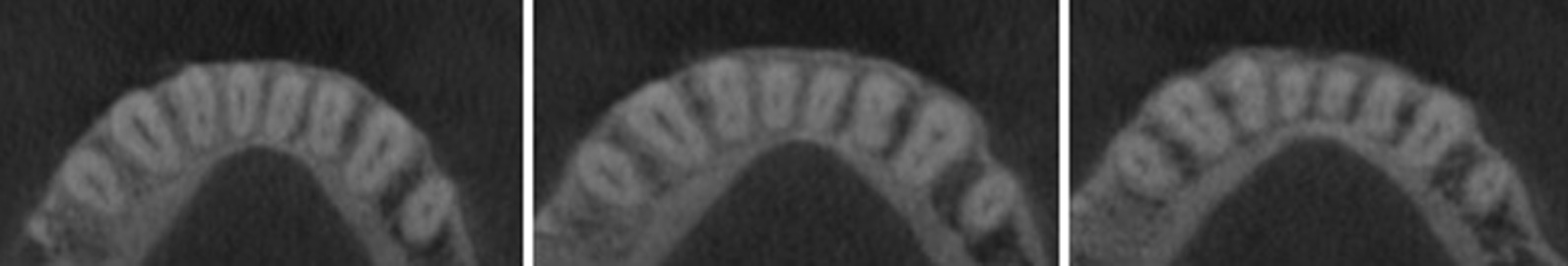
CBCT axial view of mandibular anteriors

The images were obtained with 90 kV and 10 mA as standardized operating specifications on the CBCT machine 3Dx SCANORA (Tuusula, Finland, Nahkelantie 160), and the data were collected. Both jaws were scanned with FOV = 80–100 (medium field of view), and a voxel size of 0.25 mm (standard resolution mode) was selected. The total scan time was 20 s; it included a 360° rotation of the X-ray–a receptor that surrounds the immobile patient. The images were inspected and interpreted at a workstation equipped with On-demand 3D version software 1.0.10.6388 (Daejeon, Korea, Yuseong-gu). The images are displayed on a 27-in. TFT monitor with 1280 1024 pixels of screen resolution and measured in three planes: coronal, axial and sagittal. The morphology of the root canals was determined and recorded (Fig. 1).

An expert endodontist and an observer performed the study’s calibration. In the pilot study, the observer was trained and calibrated to read the CBCT images with a sample size of 50. The observer evaluated the CBCT images using sagittal, axial and coronal views to identify the root and root canal morphology, and each tooth received a single score. Disagreements were discussed, and a consensus was reached after much deliberation.

**Fig. 2:**
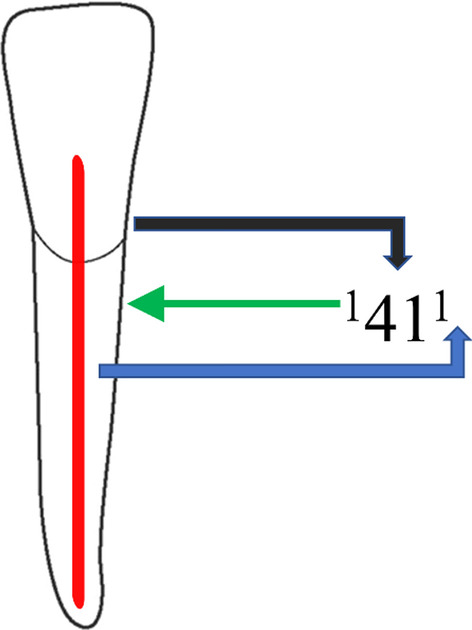
New classification system for root canal morphology of mandibular right central incisor classified using the new classification system, described as code ^1^41^1^. The code consists of three components, the tooth number—black color arrow, number of roots—green color arrow and the root canal configuration—blue color arrow. The number of roots is added as a superscript before the tooth number, so it is single root and tooth number (41). Description of root canal configuration is written as superscript after the tooth number on the course of the root canal starting from the orifices [O], passing through the canal [C], ending by the foramen [F], so it is single canal

The obtained images were divided based on the patient’s age into groups (10–20, 21–30, 31– 40, 41–50, 51–60 and 61 years above). Depending on gender, the patients were divided as male or female and based on ethnicity (Saudi, Indian, Pakistani, Egyptian, Syrian and Philippines). The root canal morphology was classified using Vertucci’s classification system and the new classification system introduced by Ahmed et al. (Fig. 2), and the difference concerning age, gender and ethnicity was recorded [21, 22].

### Statistical analysis

The SPSS software 26th version (Armonk, NY, USA, IBM SPSS Statistics) was used to conduct the statistical analysis as descriptive statistics, such as mean, standard deviation and frequency, were calculated. The Chi-square test was used to compare the root canal morphology in mandibular and maxillary anterior teeth and its relationship with gender, age and ethnicity of the patients; the significance level was set at 5% (*p* = 0.05).

## Results

**Fig. 3:**
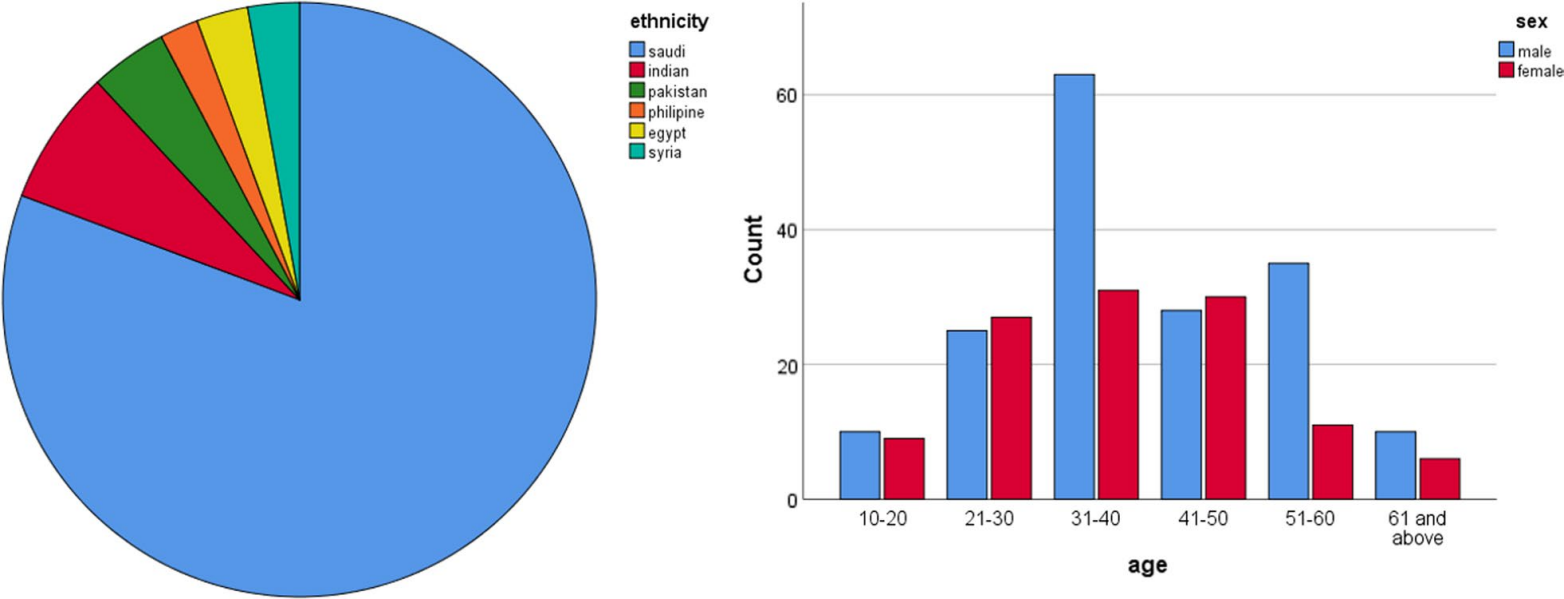
Descriptive analysis of sociodemographic characters (*n* = 285)

The characteristics of all included CBCT images are illustrated in Fig. 3. A total of 285 CBCT images of the patient were accessed through the retrospective data. The majority of samples were of Saudi nationals, and the samples consisted of 60% males and 40% females. The highest age group participating in the study was 31–40 years. According to Vertucci and the new classification systems, Table 1 shows the distribution of permanent maxillary and mandibular anterior teeth.

**Table 1:** Distribution of maxillary and mandibular anterior teeth according to Vertucci and the new classification system (NC = Non-classifiable)

Teeth position (*n* = 570)	Vertucci classification				New classification system			
	Type I	Type III	Type IV	NC	^1^TN^1^	^1^TN^1-2-1^	^1^TN^1-2^	^1^TN^1-2-1-2-1^
*Maxillary*								
Central incisors	558	12			558	12		
Lateral incisors	557	13			557	13		
Canines	559	11			559	11		
*Mandibular*								
Central incisors	489	74	7		489	74	7	
Lateral incisors	391	144	35		391	144	35	
Canines	515	40	14	1	515	40	14	1

**Fig. 4:**
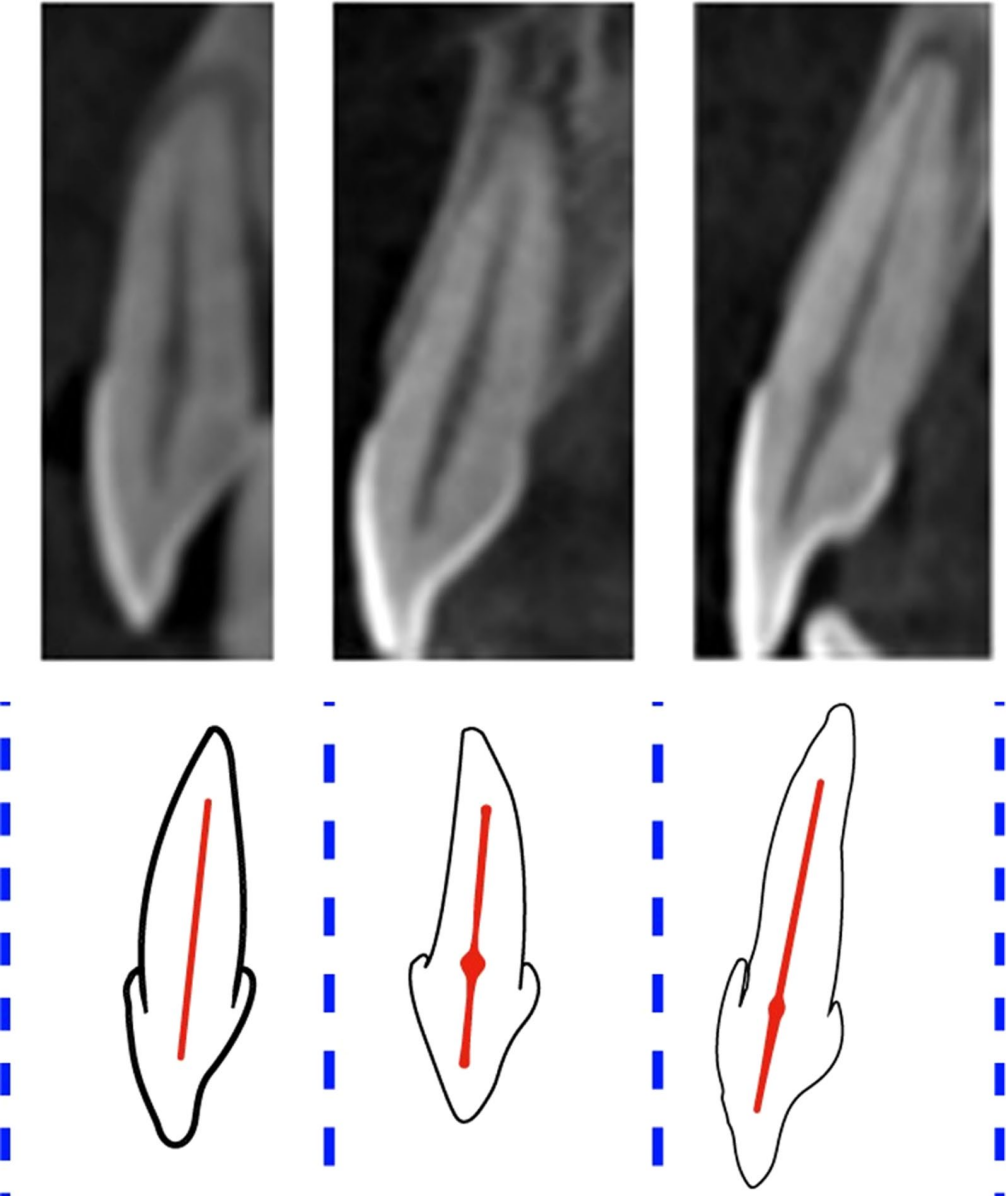
The maxillary anteriors with code ^1^TN^1^ (CBCT sagittal view)

**Fig. 5:**
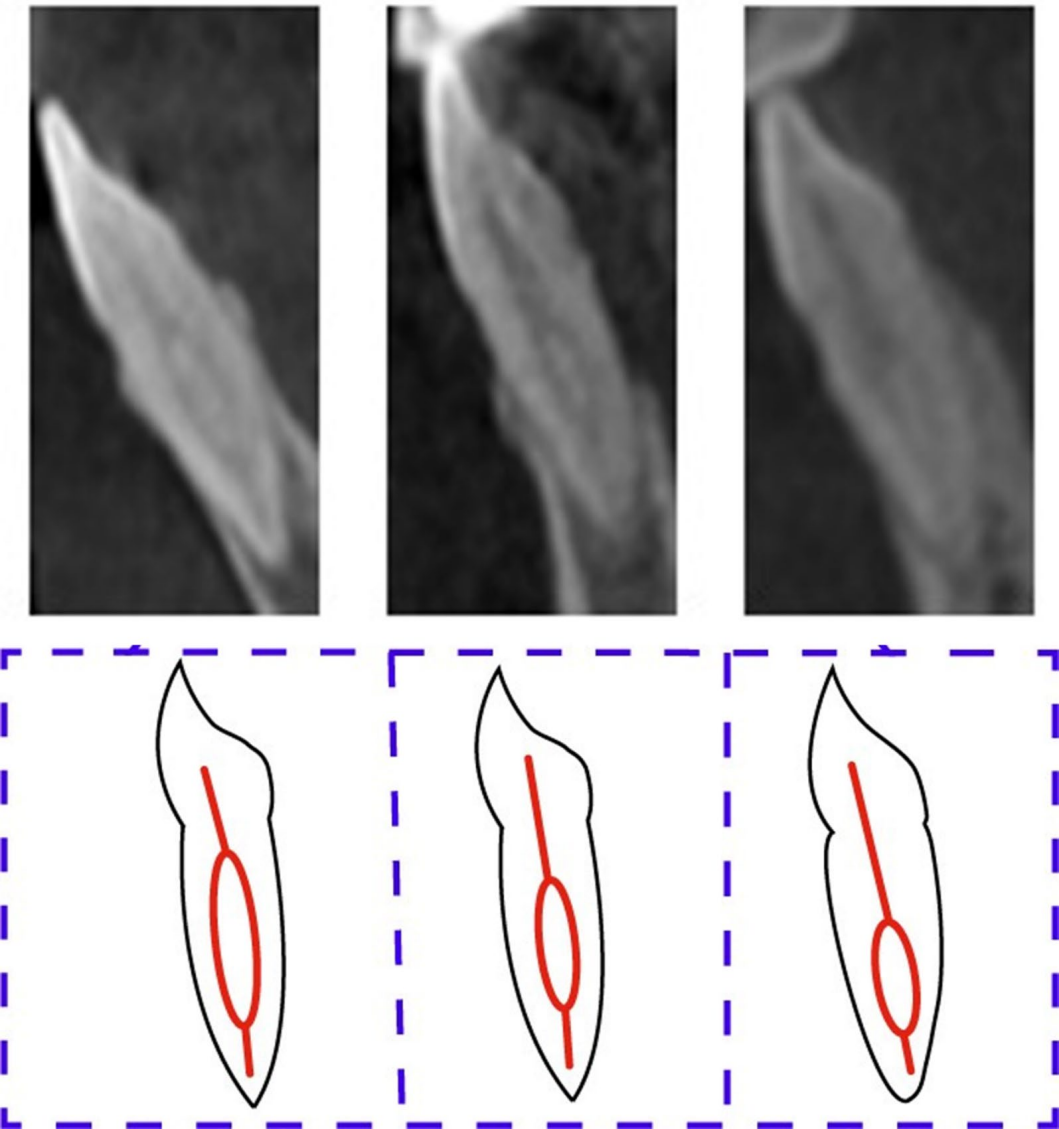
The mandibular anteriors with code ^1^TN^1-2-1^ (CBCT sagittal view)

**Fig. 6:**
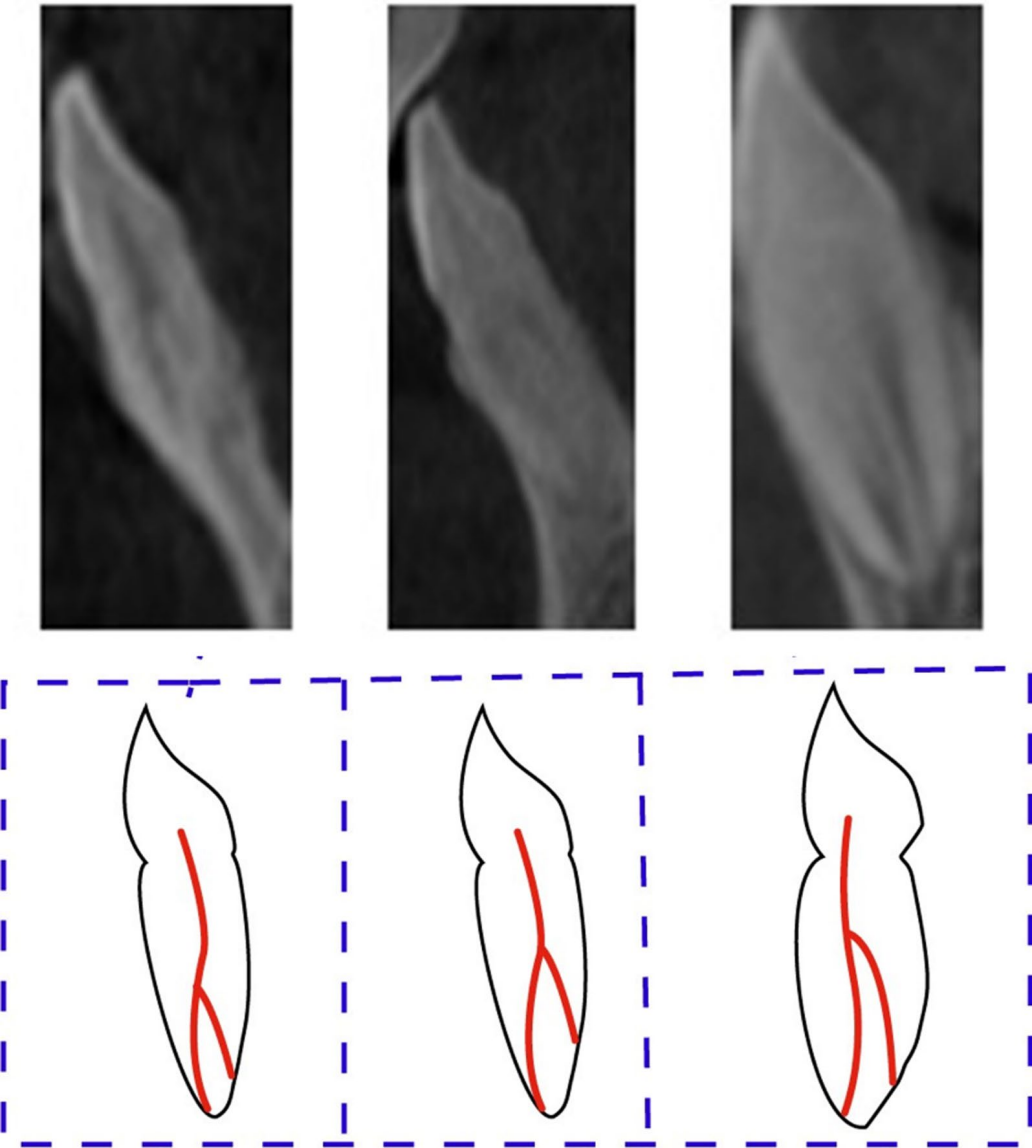
The mandibular anteriors with code ^1^TN^1-2^ (CBCT sagittal view)

**Fig. 7:**
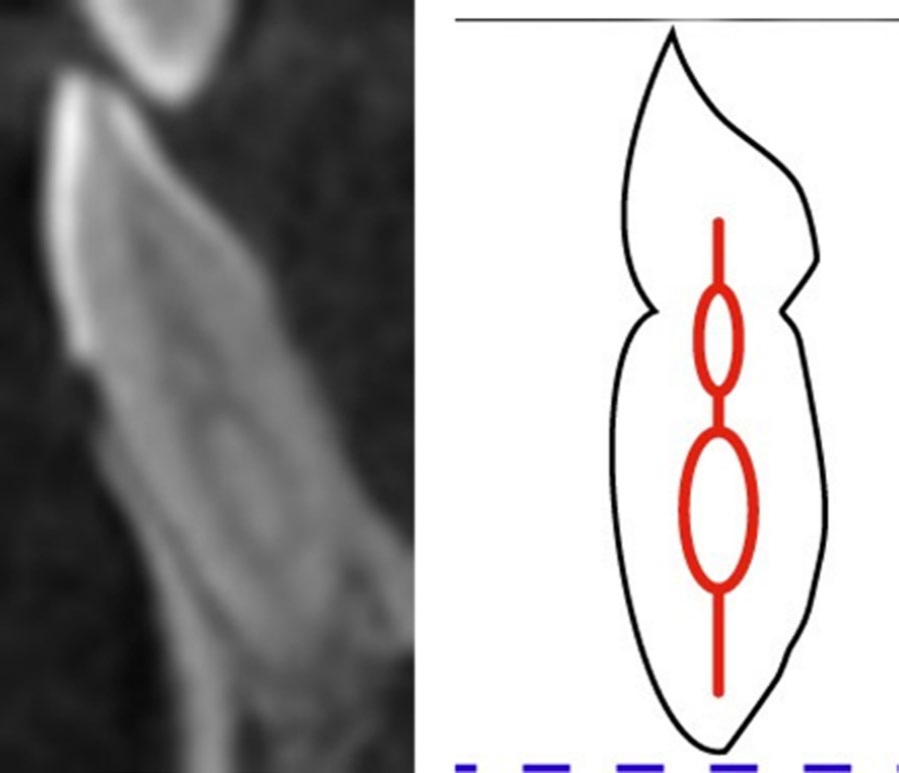
The mandibular canine with code ^1^TN^1-2-1-2^

The majority of anterior maxillary teeth was Type I of Vertucci classification and code ^1^TN^1^ of the new classification system of root canal morphology (Fig. 4). In the mandibular anterior teeth, most teeth were Type I followed by Type III and Type IV of Vertucci classification and ^1^TN^1^ followed by ^1^TN^1-2-1^ and ^1^TN^1-2^ of Ahmed classification of root canal morphology (Figs. 5, 6). A nonclassifiable type using Vertucci classification was revealed in mandibular canine; it was classified by applying the latest classification system introduced by Ahmed et al., ^1^TN^1-2-1-2-1^ (Fig. 7).

According to Vertucci classification (Table 2) and the latest classification system provided by Ahmed et al., Table 3 reveals no statistically significant difference in permanent anterior teeth except the mandibular canine, which showed a significant difference with a *p*-value < 0.05 in the relationship with gender of the patients.

**Table 2:** The root canal morphology in mandibular and maxillary anterior teeth and its relationship with gender according to Vertucci classification

Teeth position	Gender (*n* = 570)	Vertucci classification				Chi square value	(d)	*p*-value
		Type I	Type III	Type IV	NC			
*Maxillary*								
Central incisors	M	336	6			0.522	1	0.470
	F	222	6					
Lateral incisors	M	335	7			0.024	1	0.876
	F	222	6					
Canines	M	337	6			0.772	1	0.380
	F	222	5					
*Mandibular*								
Central incisors	M	296	44	4		0.865	2	0.649
	F	193	30	3				
Lateral incisors	M	253	74	19		4.977	2	0.083
	F	138	70	16				
Canines	M	320	8	10	1	12.74	2	0.002*
	F	195	32	4				

Significant value < 0.05; Cross Tab, Chi Square Test

M, male; F, female; d, difference; NC, Non classifiable

**Table 3:** The root canal morphology in mandibular and maxillary anterior teeth and its relationship with gender according to Ahmed classification

Teeth position	Gender (*n* = 570)	Ahmed classification				Chi-square value	(d)	*p*-value
		^1^TN^1^	^1^TN^1-2-1^	^1^TN^1−2^	^1^TN^1-2-1-2-1^			
*Maxillary*								
Central incisors	M	336	6			1.000 ^f^	1	0.635
	F	222	6					
Lateral incisors	M	335	7			0.024	1	0.876
	F	222	6					
Canines	M	337	6			1.000 ^f^	1	0.635
	F	222	5					
*Mandibular*								
Central incisors	M	296	44	4		0.850	2	0.654
	F	193	30	3				
Lateral incisors	M	253	74	19		4.977	2	0.083
	F	138	70	16				
Canines	M	320	8	10	1	12.74	2	0.002*
	F	195	32	4				

Significant value < 0.05; Cross Tab, Chi Square Test

M, male; F, female; TN, tooth number; f, fisher exact test; d, difference

According to the classification provided by Vertucci (Table 4) and the latest classification system provided by Ahmed et al., Table 5 revealed no significant difference concerning maxillary central incisors and canines. However, a statistically significant difference was reported in maxillary lateral incisors and mandibular anterior teeth with a *p*-value < 0.05 in the relationship between ethnicity and permanent anterior teeth root canal morphology.

**Table 4:** The root canal morphology in mandibular and maxillary anterior teeth and its relationship with ethnicity according to Vertucci classification

Teeth position	Ethnicity *(n* = 570)	Vertucci classification				Chi square value	(d)	p-value
		Type I	Type III	Type IV	NC			
*Maxillary*								
Central incisors	Saudi	452	9					
	Indian	40	2					
	Pakistani	22	1			2.993	5	0.701
	Egyptian	16	0					
	Syrian	16	0					
	Philippines	12	0					
Lateral incisors	Saudi	454	8					
	Indian	42	3					
	Pakistani	17	2			50.111	5	0.001*
	Egyptian	16	0					
	Syrian	16	0					
	Philippines	12	0					
Canines	Saudi	452	9					
	Indian	41	1					
	Pakistani	22	1			1.578	5	0.904
	Egyptian	16	0					
	Syrian	16	0					
	Philippines	12	0					
*Mandibular*								
Central incisors	Saudi	366	40	5				
	Indian	60	14	1				
	Pakistani	25	10	1		34.695	10	0.001*
	Egyptian	16	4	0				
	Syrian	14	4	0				
	Philippines	8	2	0				
Lateral incisors	Saudi	330	116	18				
	Indian	17	16	10				
	Pakistani	16	8	7		45.047	10	0.001*
	Egyptian	12	4	0				
	Syrian	8	0	0				
	Philippines	8	0	0				
Canines	Saudi	424	22	10	1			
	Indian	37	8	2				
	Pakistani	20	4	2		29.981	10	0.001*
	Egyptian	12	2	0				
	Syrian	14	2	0				
	Philippines	8	2	0				

Significant value < 0.05; Cross Tab, Chi Square Test

d, difference

**Table 5:** The root canal morphology in mandibular and maxillary anterior teeth and its relationship with ethnicity according to Ahmed classification

Teeth position	Ethnicity (*n* = 570)	New classification system				Chi-square value	(d)	*p*-value
		^1^TN^1^	^1^TN^1-2-1^	^1^TN^1-2^	^1^TN^1-2-1-2-1^			
*Maxillary*								
Central incisors	Saudi	452	9					
	Indian	40	2					
	Pakistani	22	1			4.016	5	0.547
	Egyptian	16	0					
	Syrian	16	0					
	Philippines	12	0					
Lateral incisors	Saudi	454	8					
	Indian	42	3					
	Pakistani	17	2			50.110	5	0.001*
	Egyptian	16	0					
	Syrian	16	0					
	Philippines	12	0					
Canines	Saudi	452	9					
	Indian	41	1					
	Pakistani	22	1			4.016	5	0.547
	Egyptian	16	0					
	Syrian	16	0					
	Philippines	12	0					
*Mandibular*								
Central incisors	Saudi	366	40	5				
	Indian	60	14	1				
	Pakistani	25	10	1		35.354	10	0.001*
	Egyptian	16	4	0				
	Syrian	14	4	0				
	Philippines	8	2	0				
Lateral incisors	Saudi	330	116	18				
	Indian	17	16	10				
	Pakistani	16	8	7		45.047	10	0.001*
	Egyptian	12	4	0				
	Syrian	8	0	0				
	Philippines	8	0	0				
Canines	Saudi	424	22	10	1			
	Indian	37	8	2				
	Pakistani	20	4	2		29.981	10	0.001*
	Egyptian	12	2	0				
	Syrian	14	2	0				
	Philippines	8	2	0				

Significant value < 0.05; Cross Tab, Chi Square Test

TN, tooth number; d-difference

According to Vertucci classification (Table 6) and the latest classification system provided by Ahmed et al., Table 7 demonstrates no significant difference concerning maxillary central incisors and canines. However, a statistically significant difference was reported in maxillary lateral incisors and mandibular anterior teeth with a *p*-value < 0.05 in the relationship with age; younger patients showed more root canal variations when compared with the older patients.

**Table 6:** The root canal morphology in mandibular and maxillary anterior teeth and its relationship with age according to Vertucci classification

Teeth position	Age (years) *(n* = 570)	Vertucci classification				Chi square value	(d)	*p*-value
		Type I	Type III	Type IV	NC			
*Maxillary*								
Central incisors	10–20	36	2					
	21–30	104	2					
	31–40	178	4			7.831	5	0.166
	41–50	118	2					
	51–60	90	2					
	60 above	32	0					
Lateral incisors	10–20	38	2					
	21–30	105	2					
	31–40	174	5			14.582	5	0.012*
	41–50	116	2					
	51–60	92	2					
	60 above	32	0					
Canines	10–20	36	2					
	21–30	104	2					
	31–40	180	4			8.106	5	0.150
	41–50	116	2					
	51–60	91	1					
	60 above	32	0					
*Mandibular*								
Central incisors	10–20	34	12	0				
	21–30	92	24	2				
	31–40	160	22	2		20.780	10	0.023*
	41–50	91	8	2				
	51–60	84	8	1				
	60 above	28	0	0				
Lateral incisors	10–20	33	4	4				
	21–30	52	52	2				
	31–40	112	52	24		45.065	10	0.001*
	41–50	104	12	1				
	51–60	62	20	4				
	60 above	28	4	0				
Canines	10–20	26	12	2				
	21–30	88	4	6				
	31–40	173	12	2	1	43.472	5	0.001*
	41–50	108	8	2				
	51–60	92	4	2				
	60 above	28	0	0				

Significant value < 0.05; Cross Tab, Chi Square Test

Yrs, years; d, difference

**Table 7:** The root canal morphology in mandibular and maxillary anterior teeth and its relationship with age according to Ahmed classification

Teeth position	Age (*n* = 570)	Ahmed classification				Chi-square value	(d)	*p*-value
		^1^TN^1^	^1^TN^1-2-1^	^1^TN^1−2^	^1^TN^1-2-1-2-1^			
*Maxillary*								
Central incisors	10–20	36	2					
	21–30	104	2					
	31–40	178	4			10.419	5	0.064
	41–50	118	2					
	51–60	90	2					
	60 above	32	0					
Lateral incisors	10–20	38	2					
	21–30	105	2					
	31–40	174	5			14.582	5	0.012*
	41–50	116	2					
	51–60	92	2					
	60 above	32	0					
Canines	10–20	36	2					
	21–30	104	2					
	31–40	180	4			10.419	5	0.064
	41–50	116	2					
	51–60	91	1					
	60 above	32	0					
*Mandibular*								
Central incisors	10–20	34	12	0				
	21–30	92	24	2				
	31–40	160	22	2		20.627	10	0.024*
	41–50	91	8	2				
	51–60	84	8	1				
	60 above	28	0	0				
Lateral incisors	10–20	33	4	4				
	21–30	52	52	2				
	31–40	112	52	24		45.065	10	0.001*
	41–50	104	12	1				
	51–60	60	20	4				
	60 above	28	4	0				
Canines	10–20	26	12	2				
	21–30	88	4	6				
	31–40	173	12	2	1	43.472	10	0.001*
	41–50	108	8	2				
	51–60	92	4	2				
	60 above	28	0	0				

Significant value < 0.05; Cross Tab, Chi Square Test

yrs, years; TN, tooth number; d, difference

## Discussion

This study was conducted amongst Saudi subpopulations, where majority of participants were males (60%) and Saudi nationals (80.7%) with age group 31–40 (33%). According to Vertucci and the new classification systems, our study results revealed that the prevalence of root canal variation in relation to ethnicity and age of the patients was significantly different in the maxillary laterals and mandibular anteriors, with younger patients showing more variations than older patients. The mandibular permanent anterior teeth exhibit a wide range of morphological differences in the root canal.

The motive of this study by including two different classification systems was to clarify the aberrations in question and the specific region of occurrence. The classification system by Vertucci, though being a universal one and widely known, might not provide exhaustive information about the tooth and its anomaly. Furthermore, Vertucci’s classification system does not classify the number of roots [23]. In comparison, the new classification system by Ahmed et al. provides a single code that classifies the particular tooth with the number of roots and the canal morphology [21]. In addition, it helps us understand complex intercanal communications in teeth [2].

The radiographic tool used for this study was CBCT. It possesses an array of qualities that are crucial for analysis. It is non-invasive and provides intricate external and internal details of the tooth and nearby structures [28–30]. It has a comparatively low radiation dose and is more economical than a CT scan. Moreover, we can expect almost accurate measurements given that CBCT voxels are isotropic [31, 32]. Previous studies have shown that CBCT is very reliable for demonstrating the root canal morphology and used for cross-sectional surveys, where the analysis involves a large sample size [33, 34].

The permanent anterior teeth have complex roots and canals, especially mandibular anteriors. Generally they are single-rooted; thus, we can also expect double roots or canals, apical ramification, a lateral branch of a root canal or apical furcation. These variabilities can complicate RCT for practitioners. The most regular type of configuration of root canal was Type I, followed by Type III from the Vertucci Classification, which corresponds to similar findings from other studies [4, 35]. Studies conducted among other populations, such as Israeli (89.7%), Brazilian (90.5%) and Iranian (97.6%), have reported similar findings [36–38]. All permanent anteriors under consideration had single roots, corresponding with findings from other studies [39].

Most mandibular anterior teeth configurations were Type I, followed by Type III and Type V of Vertucci classification. This finding is partly consistent with the study conducted amongst the Saudi Arabia and Indian subpopulation [40, 41]. The authors revealed the frequency of Vertucci’s Type I in mandibular laterals as most common, followed by Type III and least of all Type V. Another study showed that more than half of the mandibular centrals and laterals analysed had Type I [42, 43]. On the contrary, a study conducted by Mashyakhy showed that most mandibular incisors were Type III [44]. At the same time, studies conducted on Belgium, Iran and Chile populations showed less than 20% frequency [45–47].

Studies conducted to locate differences between root canal morphologies across different genders have shown significant differences, and similar findings were reported in this study. More variations were found in males than the samples obtained from the female population. This finding corresponds to findings obtained from similar studies conducted in Turkish and Indian populations [13, 48]. No significant difference was identified concerning maxillary canines and incisors in terms of age. However, a statistically significant difference was reported in upper lateral incisors and lower anterior teeth. A study by Lin et al. showed the insignificant difference between the age factor of the patients and the frequency of two root canals amongst the sample size [39]. Vertucci is widely applied to classify the morphology of root canal, but it has also been shown to have several deficiencies [13]. Therefore, the new system for classification by Ahmed et al. is more accurate because it offers a better demonstration of the number of roots and their canal morphology. Practitioners ought to be equipped with current advances in classification systems, the appropriate diagnostic tools and the possibilities in different anatomical aberrations. If conducted tactfully, with such skill, successful endodontic treatment can be achieved without surgical intervention.

### Limitations

The root canal morphology is capable of alteration over time. With age, secondary dentin deposition is found, thereby explaining that the results are a more significant part of the sample size above the age of 30 years. The present study investigated the root canal morphology of permanent anterior teeth in a Saudi subpopulation that used a voxel size of 0.25 mm, which is a limiting factor; it might have also been influenced by limitations of resolution as the canals become calcified and narrower. However, using a high-resolution imaging modality improves visualisation of morphology of root canal in anterior single-rooted teeth. One of the major flaws of Vertucci classification is that it does not consider the number of total roots present. Any tooth found to have more than one root is classified directly as Type IV or Type V, which is inaccurate because it would have direct implications on the endodontic procedure or placement of posts if required.

## Conclusions

Mandibular permanent anterior teeth demonstrate an extensive series of morphological difference in the root canal. Males and females did not demonstrate a wide range of variation in the morphology of root canal except for the canines. Maxillary laterals and mandibular anteriors showed the significant difference in prevalence of root canal variation in relation to the ethnicity and age of the patients. Younger patients showed more variations when compared with the older patients.

## Data Availability

The datasets used and/or analysed during the current study are available from the corresponding author on reasonable request.

## Abbreviations

CBCT: Cone-beam computed tomography
MicroCT: Micro-computed tomography
2D: Two-dimensional
3D: Three-dimensional
